# The influence of status on satisfaction with relative rewards

**DOI:** 10.3389/fpsyg.2013.00804

**Published:** 2013-10-30

**Authors:** Konstanze Albrecht, Emma von Essen, Klaus Fliessbach, Armin Falk

**Affiliations:** ^1^Faculty of Arts and Humanities, Institute of Psychology, RWTH Aachen UniversityAachen, Germany; ^2^Department of Economics and Business, Aarhus UniversityAarhus, Denmark; ^3^Center for Economics and Neuroscience, University of BonnBonn, Germany; ^4^German Center for Neurodegenerative Diseases (DZNE)Bonn, Germany; ^5^Department of Psychiatry and PsychotherapyBonn Medical School, Bonn Germany; ^6^Department of Economics, Institute for Applied Microeconomics, University of BonnBonn, Germany

**Keywords:** status, social comparison, relative reward, satisfaction, fairness

## Abstract

This study investigates how induced relative status affects satisfaction with different relative payoffs. We find that participants with lower status are more satisfied with disadvantageous payoff inequalities than equal or higher status participants. In contrast, when receiving an advantageous payoff, status does not affect satisfaction. Our findings suggest that relative social status has important implications for the acceptance of income inequalities.

## Introduction

Income is considered an important factor for job satisfaction (Clark and Oswald, 1996; Boyce et al., 2010). But how do people arrive at valuations of income? According to previous literature, two main factors influence this evaluation process: Self-serving preferences and fairness considerations (e.g., Adams, 1965; Diekmann et al., 1997; Moore and Loewenstein, 2004; van den Bos et al., 2006).

Self-serving preferences are based on what pleases people and what they evaluate positively, as for example a salary increase (Messick and Sentis, 1979, 1983; van den Bos et al., 2006). Fairness considerations, on the other hand, take into account the outcomes of other people, as for example a colleague's salary (Adams, 1965; Fehr and Schmidt, 1999; Falk and Fischbacher, 2006). I may prefer a higher to a lower salary, but at the same time consider it unfair to receive more than my colleague who does the same job. In this case, a self-serving preference for money collides with beliefs about what is fair and right: Satisfaction with a salary increase is affected positively because more money is preferred to less money, but it is also affected negatively, because it is considered unfair to receive more than a colleague (van den Bos et al., 2006).

If we consider an example in which my colleague and not I would receive a salary increase although we do the same work, I might regard the situation as both non-self-serving *and* unfair. Thus, there would be two sources of negative affect which both reduce my satisfaction with this outcome (van den Bos et al., 1997, 1998, 2006).

A number of studies show that, accordingly, satisfaction with advantageous inequity (i.e., receiving more money than another person) is higher than satisfaction with disadvantageous inequity (i.e., receiving less money than another person); (Buunk and van Yperen, 1989; van den Bos et al., 1997, 1998, 2006; Peters et al., 2008). In line with this, when both my colleague and I would receive a salary increase for the same work, I should be more satisfied than in either of the two aforementioned examples: I receive money *and* the monetary allocation is fair (e.g., Fehr and Schmidt, 1999; Falk and Fischbacher, 2006; van den Bos et al., 2006).

So far, only a few studies have investigated the influence of social context, such as the relationship to the person with whom the income comparison takes place (Loewenstein et al., 1989; Gächter and Riedl, 2006; Peters and van den Bos, 2008; Peters et al., 2008). Loewenstein et al. (1989) showed that this relationship indeed does affect satisfaction: In friendly relationships, people disliked receiving a higher payoff than their opponent. When the relationship was antagonistic, people did not care about the payoff of their opponent, as long as it was less than or equal to their own. Results by Peters et al. (2008) support these findings: In their study, people were less satisfied with advantageous inequity when their opponent was a close friend compared to a stranger. This suggests that people take social context into account when rating one's satisfaction, with fairness considerations playing a bigger role when the relationship with the other person is positive.

An important component of social context that only few studies have considered is social status; although it is suggested to play a crucial role in distributive justice (Lind and Tyler, 1988; Ellemers et al., 1999; Blanz et al., 2011). Social status has been shown to influence, for example, an individual's engagement in pro- and anti-social behavior (Piff et al., 2010, 2012), perceptions of deservingness (Ellemers et al., 1999; Hong and Bohnet, 2007) and even life satisfaction (Boyce et al., 2010). Several studies have further shown that status is strongly associated with increased economic benefits and entitlements. For example, high status participants have been found to receive higher outcomes in bargaining games (Ball and Eckel, 1997; Ball et al., 2001), and to be punished less for unsocial behavior (von Essen and Ranehill, 2012), than low status participants.

However, little is known about how relative status affects satisfaction with payoff allocations. The present study investigates this question systematically, by varying status (higher, same, and lower) and payoff allocations (advantageous, equal, and disadvantageous) within pairs of individuals.

In line with previous research, we expect satisfaction with advantageous inequity to be higher than satisfaction with disadvantageous inequity in general (Buunk and van Yperen, 1989; van den Bos et al., 1997, 1998, 2006; Peters et al., 2008).

Status might further moderate satisfaction in different ways. First, subjects might use status in general to justify a neglect of fairness considerations, and thereby increase their satisfaction with unequal payoff allocations. Self-serving preferences for money would then have more weight and satisfaction with advantageous payoffs would be rated higher. The direction of relative status (i.e., higher or lower) should not play a role in this case; just the fact of having *another* status than the opponent would imply a higher satisfaction (cf. ingroup–outgroup effects; Brewer, 1979; Tajfel et al., 2006; Volz et al., 2009). Second, subjects might use status to justify their satisfaction while taking the direction of relative status into account (Hinkle and Brown, 1990). In this case, a higher status entitles to more and thus, satisfaction with an advantageous payoff would be higher when one possesses a higher status, and lower when the opponent possesses a higher status. Accordingly, the satisfaction with a disadvantageous payoff would be higher when one possesses a lower status, and lower when the opponent possesses a lower status. Third, the influence of status could also be asymmetric such that subjects, when disadvantaged, take status into account in order to reduce the negative affect of receiving less money and being treated unfairly. When advantaged, subjects may be less negatively affected and hence feel no need to take status into account.

## Materials and methods

### Participants

Previous literature indicates that men's behavior is more sensitive to status than women's behavior (e.g., Huberman et al., 2004). Thus, we invited only men to participate. We ran six sessions in which in total 133 subjects (i.e., on average 22 subjects per session; mean age 25 years) participated. Participants were recruited via ORSEE (Greiner et al., 2003). The study was conducted in the BonnEconLab in Germany in accordance with institutional and national ethics guidelines and regulations. Informed consent was obtained from all participants. Participants were seated in cubicles and the experiment was presented on individual computer screens, programmed with z-Tree (Fischbacher, 2007) All participants received EUR 4 for participating. In addition one of the 15 payoff allocations, as described below, was randomly chosen for payment. Participants on average earned EUR 24 (~USD 31).

### Material and procedure

In the experiment we first induced relative status. In a second step, subjects were confronted with different payoff allocations which they had to evaluate.

Relative status was induced by means of a trivia quiz which consisted of 30 questions, each presented with four alternative answers of which only one was correct (For example: “How fast does Pluto move in its orbit around the sun? (A) 5.7 km/s, (B) 6.1 km/s, (C) 6.8 km/s or (D) 4.7,km/s.” The quiz in its full length is displayed in the Appendix.) Based on the total quiz score, participants were assigned to three different groups: a *low*, a *medium*, and a *high* score group, which served as our status groups. The four highest-scoring participants of each experimental session were allocated to the high score group, the four lowest scoring participants were allocated to the low score group, and the remaining participants were allocated to the medium score group. Accordingly, the medium score group consisted of the 16 to 20 participants per session who scored lower than the high score group but higher than the low score group; and who scored similarly as participants of their own group. In total, 24 subjects were allocated to the high and the low score groups respectively, whereas the medium score group comprised 85 participants. Thus, we could fully vary status in all directions within individuals in the medium score group, i.e., subjects from this group would face subjects from a higher scoring group, subjects from their own group, and subjects from a lower scoring group. Participants were aware of how groups were constructed, although the word “status” was not mentioned to them. (Instructions are provided in the Appendix).

In order to manipulate social status in a laboratory setting, previous studies have used random assignments to high and low status groups, as well as assignments using scores from trivia quiz. Scores from a trivia quiz have been used to induce status that was—or that participants believed to be—dependent on ability (Ball et al., 2001; Gächter and Riedl, 2006). In a direct comparison of both assignment procedures, Ball et al. (2001) found similar results for both random status and status participants believed to be ability-dependent: Participants with higher status earned more in a bargaining game than participants with lower status. In our experiment, we use a difficult multiple choice quiz to create a status based on a mixture of ability (knowing the correct answers) and chance (guessing the correct answers). We consider this is an appropriate way to induce status, given that, when acquiring status, both ability and coincidence possibly play an important role.

In the second part of the study, which followed right after completion of the first, we measured subjects' satisfaction with different payoff allocations between themselves and other subjects. Each subject was presented with a series of payoff allocations, and asked to rate each allocation on a scale ranging from −5 to +5. More specifically, participants were asked: “How happy are you with the following payoff allocation between you and the other participant?” For each allocation, a star indicated who scored higher on the quiz. If no star appeared both participants belonged to the same score group. We used the star, since this symbol is commonly associated with a higher rank; for instance in military, or in the hotel and restaurant businesses (cp. Zink et al., 2008). Participants were explicitly told that “the size of the payoff is independent of the quiz result.”

In total, all participants were presented with the same 15 payoff allocations in random order. Only in the medium score group (intermediate status group), subjects were presented with allocations between themselves and higher, same, as well as lower status group subjects. Subjects from the high score group (high status group) and the low score group (low status group) were only presented with payoff allocations between themselves and lower or higher status participants, respectively. They are analyzed separately, serving as a robustness check for our main results. In our main analyses, we concentrate on the intermediate status group and herein on the allocations in which the participant himself always received EUR 20 (Table 1). We do so in order to hold absolute own payoff constant and hence avoid effects of absolute income. This yields two factors (*Status* and *Payoff Allocation)* and allows us to compare all possible combinations between higher, same and lower status and advantageous, equitable and disadvantageous payoff allocations, respectively.

**Table 1:** **Payoff allocations in EUR**.

**Social status information**	**Payoffs (D)**	**Payoffs (E)**	**Payoffs (A)**	**Payoffs (D)**	**Payoffs (A)**
You:Him^*^(lower status)	**20:30**	**20:20**	**20:10**	10:20	30:20
You:Him (same status)	**20:30**	**20:20**	**20:10**	10:20	30:20
You^*^:Him (higher status)	**20:30**	**20:20**	**20:10**	10:20	30:20

D, disadvantageous; A, advantageous; E, equitable.

* For each allocation, a star indicated who scored higher on the quiz. Only allocations in which the subject himself received EUR 20 (boldface) were entered in the analyses. To prevent subjects from repeatedly seeing the exact same monetary amounts, we varied the rewards within a 10% interval from the mean.

## Results

Our variable of interest is the satisfaction rating of individuals from the intermediate group since only this group faced opponents from all three status groups. (See Methods section; analyses of the high and low status groups can be found in the Appendix. We find a similar result pattern as for the intermediate status groups).

We conducted an ANOVA with the two within-subject factors *Status* (lower, same, higher) and *Payoff Allocation* (disadvantageous, equitable, advantageous). Significant main and interaction effects of these factors were further studied with pair-wise comparisons (Sidak-corrected for multiple comparisons).

**Figure 1:**
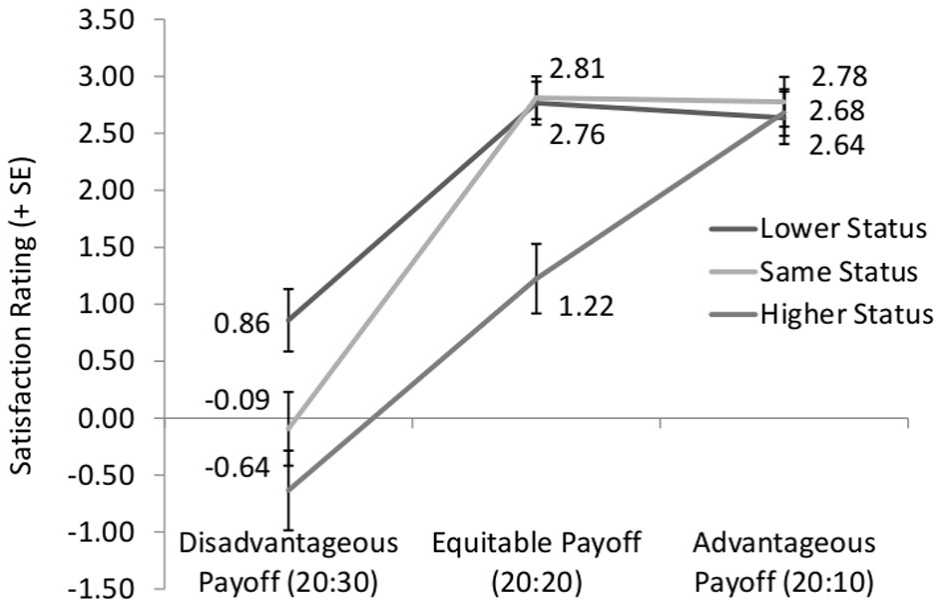
**The *x*-axis displays the relative payoffs and the *y*-axis displays average satisfaction ratings concerning these relative payoffs**.

The ANOVA yields a significant main effect of Status [*F*_(1.785, 149.947)_ = 29.724, *p* < 0.001, partial η^2^ = 0.261], a significant main effect of Payoff Allocation [*F*_(1.500, 125.961)_ = 62.652, *p* < 0.001, partial η^2^ = 0.427], and an interaction effect of the two factors [*F*_(3.390, 284.742)_ = 10.143, *p* < 0.001, partial η^2^ = 0.108]. Results of the pair-wise comparisons (corrected for multiple comparisons) are displayed in Table 2 and are discussed below. Figure 1 provides an overview of the results, including means and standard errors (SE).

**Table 2:** ***P*-values of pairwise comparisons (Sidak-corrected for multiple comparisons) of satisfaction ratings between different payoff allocations and different status categories**.

**Social status information**	**Satisfaction D-E**	**Satisfaction D-A**	**Satisfaction E-A**
	***P***	***P***	***P***
Lower status	**<0.001**	**<0.001**	0.950
Same status	**<0.001**	**<0.001**	0.998
Higher status	**<0.001**	**<0.001**	**<0.001**
**Satisfaction with**	**Lower-same status**	**Lower-higher status**	**Same-higher status**
	***P***	***P***	***P***
Payoff (D)	**0.001**	**<0.001**	**0.060**
Payoff (E)	0.990	**<0.001**	**<0.001**
Payoff (A)	0.887	0.994	0.948

D, disadvantageous; A, advantageous; E, equitable. Significant results (p < 0.05) are in boldface.

### Satisfaction with payoff allocations across status categories

As Figure 1 shows, disadvantageous payoffs are rated as less satisfactory than advantageous and equitable payoffs, irrespective of relative status [*F*_(1.407, 59.104)_ = 27.566, *p* < 0.001, partial η^2^ = 0.396] Pair-wise comparisons of the payoffs (Sidak-corrected for multiple comparisons) yield significant differences between both disadvantageous and advantageous (*p* < 0.001) as well as disadvantageous and equitable payoffs (*p* < 0.001). Yet, equitable payoffs are not rated as more satisfactory than advantageous payoffs (Sidak-corrected pairwise comparision, *p* = 0.588). Table 2 shows that this is also the case when taking into account satisfaction ratings of the same status category (which should be closest to social comparison studies without a status manipulation) only.

### The influence of status on satisfaction with different payoff allocations

The influence of relative status on satisfaction ratings shows a striking asymmetry: While status affects satisfaction when payoffs are disadvantageous, it does not affect satisfaction when payoffs are advantageous (see Figure 1 and Table 2 for statistics). Satisfaction ratings for disadvantageous inequity differ between all status categories, with highest ratings when subjects are in the lower status category and lowest ratings when they are in the higher status category.

Figure 1 further shows that for equitable payoffs, ratings do not differ between the same and the lower status categories, but are lower when participants are in the higher status category.

### The influence of status on satisfaction across payoff allocations

When we further compare satisfaction ratings across relative payoff allocations, we find that individuals with lower status are, in general, more satisfied (mean: 2.09) than higher status individuals (mean: 1.09); [*F*_(1, 84)_ = 42.420; *p* < 0.001, partial η^2^ = 0.336].

## Discussion

In the present study, we investigated the influence of status on satisfaction with different payoff allocations. In this section, we will first discuss the overall effect of payoff allocations on satisfaction across status categories. Second, we will discuss the asymmetric influence of status; while it affects satisfaction when payoffs are disadvantageous, it does not seem to affect status when payoffs are advantageous. Concluding, we will discuss the finding that satisfaction with payoffs in general is higher when subjects are of lower compared to higher status.

### Satisfaction with payoff allocations across status categories

Disadvantageous payoffs are rated as less satisfactory than advantageous and equitable payoffs, irrespective of relative status. This finding supports previous studies showing that equitable payoffs are preferred to disadvantageous payoffs (e.g., Loewenstein et al., 1989) and that fairness considerations are asymmetric; disadvantageous inequity is less satisfactory than advantageous inequity (e.g., Buunk and van Yperen, 1989; van den Bos et al., 2006; Peters and van den Bos, 2008). In contrast, equitable payoffs are not rated as more satisfactory than advantageous payoffs; this is the case across status categories as well as within the same status category only. There is evidence in the literature supporting this finding (e.g., Austin et al., 1980), but there also exists contradicting evidence showing that equitable payoffs are more satisfactory than advantageous payoffs (e.g., Buunk and van Yperen, 1989; van den Bos et al., 2006). Yet, our result is in line with the assumption that receiving *more* than another person is valued more positively than it is considered unfair. It has been shown that people prefer downward comparisons, i.e., it makes them feel better to compare themselves to people who are worse off (Festinger, 1954; Wills, 1981). Further, satisfaction seems to be higher the higher one's income is compared to the income of others (Clark and Oswald, 1996; Boyce et al., 2010). A neuroimaging study by Fliessbach et al. (2007) further supports this suggestion; they find activation in the nucleus accumbens, a brain area associated with subjective value encoding, to be positively related to the ratio between a person's own income and other's income.

### The influence of status on satisfaction with different payoff allocations

The influence of relative status on satisfaction ratings supports our third hypothesis since it shows a striking asymmetry: While status affects satisfaction when payoffs are disadvantageous, it does not affect satisfaction when payoffs are advantageous. Subjects might feel entitled to less than another person when they have lower status and thus are more satisfied. However, this seems not to be the case when subjects are advantaged.

A possible explanation is that receiving a lower relative payoff, which is neither self-serving nor fair, triggers a negative affect, which can be reduced by taking status into account. Yet, receiving a higher payoff does not trigger a negative affect since it serves an individual's self-interest. Thus, status is not taken into account when receiving a higher payoff.

Ratings of equitable payoff allocations stand in contrast to both ratings of disadvantageous and advantageous payoffs: Ratings do not differ between the same and the lower status category, but are lower in the higher status category. This suggests that participants do ignore status information when it is negative (i.e., when they have lower status), but feel entitled to more when status information is positive (i.e., when they have higher status). A possible explanation could be that people tend to integrate positive information better than negative information (see literature on selective perception/the good-news–bad-news effect, e.g., Bradley, 1978; Lord et al., 1979; Babcock and Loewenstein, 1997; Eil and Rao, 2011; Sharot et al., 2012).

Ingroup effects might also play a role; same status participants might be considered the ingroup, whereas participants with higher or lower status might be considered outgroup members. Empirical studies show that an ingroup is favored over an outgroup when it comes to the distribution of rewards or the evaluation of the groups' characteristics (e.g., Brewer, 1979; Tajfel et al., 2006; Volz et al., 2009). However, in our study in-/outgroup effects cannot fully explain the results, since satisfaction ratings are asymmetric: Satisfaction in comparisons to lower status subjects differs from satisfaction in comparisons to higher status subjects. This indicates that status affects satisfaction beyond manipulating mere group belonging.

### The influence of status on satisfaction across payoff allocations

The finding that individuals are in general more satisfied with their payoffs when they have lower compared to higher status suggests that being of inferior social status yields a higher acceptance of disadvantageous income inequalities.

The reasons for why lower status leads to higher payoff satisfaction are manifold. In a seminal article, Keltner et al. (2003) discuss the effects of power on psychological variables, such as affect, prosociality and sensitivity for reward. Although power is not the same as status, the constructs are related (e.g., French and Raven, 1959) and might have similar effects. Thus, it is possible that having a high status in our study leads to a high need for reward and accordingly to lower satisfaction ratings for disadvantageous payoffs. Further, a high status might decrease prosociality and fairness considerations, also leading to a negative effect on satisfaction. Yet the relationship between power and status in this study remains speculative. Further, in our study, status was varied within subject; hence, the aforementioned effects of status would have had to occur within person over the course of less than half an hour. Nevertheless, we cannot rule out that status affects the aforementioned variables. Future research is necessary to discover the reasons for differences in satisfaction ratings.

### Potential limitations of our study

Although our setup has the advantage of varying status within subject in a controlled laboratory setting, we acknowledge that the artificial nature of this procedure might limit its interpretability with regard to the effects of real-life status. It would therefore be highly interesting to investigate the influence of real-life status differences on payoff satisfaction in the field and compare these findings to the results from controlled laboratory studies as the present one. This would combine the advantages of both approaches and hence increase the interpretability of the present data.

As another consequence of our experimental setting, we cannot completely rule out an experimenter demand effect, since status information was explicitly given by displaying an asterisk and thus might have led subjects to assume that the experimenter wanted them to incorporate this information in their satisfaction ratings. However, such an effect is unlikely to have occurred for two reasons. First, subjects were explicitly informed that payoffs were *not* related to performance. Second, the results showed a strong asymmetry. If an experimenter demand effect would have occurred, we would have expected it to be symmetric; i.e., that status has an effect on satisfaction ratings not only when subjects are disadvantaged, but also when they are advantaged.

In our study, status was derived from some sort of performance (knowledge) measure and subjects were informed that payoffs were not related to performance. Furthermore, questions in our quiz were designed in a way that accuracy depended on luck more than on actual ability. However, we cannot rule out that subjects may have believed that payoffs were justified by differences in performance. Previous studies suggest that this most likely has no impact, because they have shown that, irrespective of whether status depended on performance or luck, higher status individuals earned more money during a bargaining game than lower status subjects (Ball and Eckel, 1997; Ball et al., 2001). Nevertheless, we suggest that future research should address the impact of status information which is derived from a completely arbitrary measure (such as dice rolls) or from highly ability related measures in different contexts.

Further, since satisfaction was measured via self-reports, there is a possibility that subjects may have not revealed their true feelings. Thus, instead of actually being more satisfied, lower status people may simply have felt not entitled to complain as much as higher status people. Accordingly, lower status people might have stated to be more satisfied than they actually were. To reduce the chance that subjects would not reveal their actual preferences, future studies could follow up on Ball and Eckel (1997) and use monetary incentives; e.g., by letting higher and lower status subjects play bargaining games and thus let behavior affect payoffs. Further, neuroscientific investigations could shed light on the influence of status on actual payoff preferences by identifying the neural correlates of the underlying cognitive processes instead of relying on self-reports.

## Concluding remarks

Altogether, our findings may not fully explain, but contribute to an explanation of why economic inequality is often accepted and persists in most societies.

Our results further highlight an important shortcoming of existing research on relative income; the study of social context in terms of status. Future research should include status and other types of social context in empirical studies and in psychological and economic models of social preferences such as equity and fairness concerns (Adams, 1965; Fehr and Schmidt, 1999; Falk and Fischbacher, 2006).

## Author contributions

Konstanze Albrecht, Emma von Essen, Klaus Fliessbach, and Armin Falk prepared the experiment, Konstanze Albrecht conducted the experiment, Konstanze Albrecht and Emma von Essen analyzed the data, and Konstanze Albrecht, Emma von Essen, Klaus Fliessbach, and Armin Falk wrote the manuscript.

### Conflict of interest statement

The authors declare that the research was conducted in the absence of any commercial or financial relationships that could be construed as a potential conflict of interest.

## Appendix

### A1. TRIVIA QUIZ

1. How long is the longest highway in the world?
  7000 miles
  8000 miles
  9000 miles
  6000 miles
2. How long is the English channel?
  612 kilometers
  564 kilometers
  551 kilometers
  627 kilometers
3. Where do you find the largest bell in the world?
  Tsar Kolokol, Kremlin, MoscowGreat Bell of Kyoto, Chion-In Temple, Kyoto
  Liberty Bell, Liberty Bell Center, Philadelphia
  Big Ben, St. Stephen's Tower, London
4. Which country is the primary producer of news print in the world?
  USA
  Great Britain
  Japan
  Canada
5. The Vinson Massif is the highest mountain of which continent?
  Antarctica
  North America
  Australia
  Africa
6. World record speed attained in a helicopter?
  239 mph
  198 mph
  249 mph
  158 mph
7. How much did it cost to run the BBC website 2002?
  72 million Pound
  65 million Pound
  59 million Pound
  48 million Pound
8. The royal mint is recognized as the oldest established business in the UK—what year?
  1886
  1897
  1901
  1902
9. How many nuclear reactors are there in the UK?
  35
  40
  45
  50
10. In post World War II general elections, in which year did the Conservative Party in the United Kingdom achieve its highest ever number of votes?
  1989
  1990
  1991
  1992
11. In what year was 3rd Class rail travel abolished in the UK ?
  1958
  1957
  1956
  1955
12. Braxy is a fatal bacterial infection in which animal?
  Cow
  Horse
  Sheep
  Pig
13. Who became president of Uganda after Idi Amin was overthrown in 1980?
  Milton Obote.
  BenedictoKiwanuka
  Paulo Muwanga
  Tito Okello
14. How much water falls down the 54 m high Niagara falls per second?
  1000 m^3^
  1500 m^3^
  2000 m^3^
  2500 m^3^
15. How fast does Pluto move in its orbit around the sun?
  4,7 km/s
  5.7 km/s
  6.1 km/s
  6.8 km/s
16. The group ABBA is one of Swedens most wellknown pop groups. How many records have they sold by now?
  300 million
  400 million
  500 million
  600 million
17. Which of these animals walk like a camel?
  The cat
  The dog
  The sheep
  The horse
18. Which country is the world's leading egg producer?
  Japan
  China
  Vietnam
  Korea
19. Which of these countries have most tractors per capita?
  Iceland
  Canada
  Japan
  Australia
20. What is the least popular month for U.S. weddings?
  January
  February
  November
  December
21. How many days does a cat usually stay in heat?
  Five
  Six
  Seven
  Eight
22. How many known poisonous birds are there in the world?
  None
  One
  Two
  Three
23. What's the first word uttered in film Citizen Kane?
  Rosebud
  Xanadu
  Kane
  Susan
24. What European nation consumes more spicy Mexican food than any other?
  Sweden
  Finland
  Denmark
  Norway
25. Buckminster Fuller's innovative inventor's Dymaxion car could carry eleven passengers, exceed 120 mph and get 30 miles per gallon in what year was this?
  1937
  1934
  1928
  1927
26. In what year did the boxer George Foreman make his first title defense in 21 years?
  1994
  1995
  1996
  1997
27. How hot is the core of the sun, in degrees Farenheit?
  32,000,000 degrees Fahrenheit
  27,000,000 degrees Fahrenheit
  20,000,000 degrees Fahrenheit
  18,000,000 degrees Fahrenheit
28. In what year did London loose 4,000 people to a “killer fog” of carbon dioxide?
  1949
  1952
  1954
  1955
29. How many days can an ant survive under water?
  One
  Two
  Three
  Four
30. What is the maximum flight speed of a Boeing 747–300 jetliner?
  611 miles per hour
  599 miles per hour
  583 miles per hour
  488 miles per hour

How much does the Eiffel tower weigh in tons? _________^1^

### A2: results of high and low status groups

Table A1 shows the effect of status and relative income on satisfaction in the high and low status groups.

### A3: instructions (stage 1)

Thanks for participating in this study in economics!

Please read the following instructions carefully. They contain everything you need to know in order to participate in the study. If you have any questions after having read the text below, please raise your hand and we will come to you.

You will be presented with 40 questions one after the other on the computer screen. These questions could be perceived as difficult, but we ask you to think it through and give each question a try. In case you have no idea, make your best guess and choose one of the alternatives. Most people will not know the answer to any of these questions, but have to guess. To answer a question you simply click on the alternative that you think is correct. The next question will then appear automatically. There is no time limit for answering a question, so you can take as much time you need for answering each question.

Please wait after you have finished all questions. The second part of today's study will start soon.

### A4: instructions (stage 2)

Please read the following instructions carefully. They contain everything you need to know for your participation. Should you have any questions after having read the text below please raise your hand and we will come to you to answer your question.

**Table A1:** **Mean satisfaction ratings and *>p*-values in high status group (i.e., Subjects face lower status only)**.

**Social status information**	**Mean satisfaction**			**Satisfaction D-E**		**Satisfaction D-A**		**Satisfaction E-A**	
	***M_D_***	***M_E_***	***M_A_***	***T***	***p***	***T***	***p***	***T***	***p***
Higher status	−1.63	0.96	2.42	−5.327	<0.001	−5.769	<0.001	−1.904	0.070

Degrees of freedom (df) = 23 for all comparisons. D, disadvantageous; A, advantageous; E, equitable.

**Table A2:** **Mean satisfaction ratings and *p*-values in low status group (i.e., Subjects face higher status only)**.

**Social status information**	**Mean satisfaction**			**Satisfaction D-E**		**Satisfaction D-A**		**Satisfaction E-A**	
	***M_D_***	***M_E_***	***M_A_***	***T***	***p***	***T***	***p***	***T***	***p***
Lower status	1.83	2.88	2.92	−1.894	0.071	−1.919	0.067	−0.095	0.925

Degrees of freedom (df) = 23 for all comparisons. D, disadvantageous; A, advantageous; E, equitable.

Now, in a number of settings, you will be randomly paired with different persons who also participated in the questionnaire in part 1 of this study. In each trial you will be presented with monetary amounts for you and the other person.

In detail you will, before every setting, be asked to click on a gray rectangle. Through this procedure it will be randomly determined what other person will be your counterpart in the trial, and consecutively how much you and this other person will receive. The size of the payoff is independent of the quiz result.

As soon as you clicked on the rectangle, it will become clear who are playing with. For instance, it may then read: “Please press a button to see the amount the other person^*^ will receive and the amount you will receive.” A star is used to indicate the player who scored higher in the quiz. When, as in this example, a star appears behind “the other person,” it means that he had a higher score than you on the quiz. If, on the other hand, the star appears after the “you,” it means that you were the player with the higher score. When there is neither an asterisk behind “the other person” nor behind the “you,” it means that you both performed about equally well on the quiz. When you will press a button now, two amounts will be presented, one will be allotted to the other person, and one will be allotted to you.

The stars are presented according to the quiz score. There will be three groups. One group consists of the four participants with the highest scores. Another group consists of the four participants with the lowest scores. The remaining participants, whose scores are between those of the four best and four worst participants, also form a group. So when there is no star displayed, it means you and your opponent are in the same group. When there is a star behind “the other person,” this means that your opponent is from a better group. A star behind “you” means that your opponent is from a worse group.

Afterwards, you will be asked to press a button. After you did this, two monetary rewards are displayed. One of the rewards will be for the other person and one will be for you. Then a scale will be presented on which you shall evaluate how satisfied you were with the allocation of the amounts. If you choose “−5,” this means you are very unsatisfied, a “0” means you are neither satisfied nor unsatisfied, and an “+5” means you are very satisfied. Other numbers accordingly mean an in-between value on the scale.

This is how the scale looks:

This session will last approximately 20 min.

After the study is over, one of the trials you faced will be randomly chosen. You will be paid according to the outcome in this trial. The drawing of the trial that will actually be paid is random and independent of how satisfied you were. The other person of this trial will also receive money according to the amount which was displayed next to his initials. The draw will be random, and although it is possible that “the other person” presented to you is repeatedly the same, it will be guaranteed that every person, including you, will only receive one payment.

If you have any questions, please ask them now. Otherwise please wait until we will tell you when to start.
